# Sunlight-Sensitive Anti-Fouling Nanostructured TiO_2_ coated Cu Meshes for Ultrafast Oily Water Treatment

**DOI:** 10.1038/srep25414

**Published:** 2016-05-10

**Authors:** HaoRan Liu, Aikifa Raza, Abulimiti Aili, JinYou Lu, Amal AlGhaferi, TieJun Zhang

**Affiliations:** 1 Department of Mechanical and Materials Engineering, Masdar Institute of Science and Technology, P.O. Box 54224, Abu Dhabi, UAE

## Abstract

Nanostructured materials with desired wettability and optical property can play an important role in reducing the energy consumption of oily water treatment technologies. For effective oily water treatment, membrane materials with high strength, sunlight-sensitive anti-fouling, relative low fabrication cost, and controllable wettability are being explored. In the proposed oily water treatment approach, nanostructured TiO_2_-coated copper (TNS-Cu) meshes are used. These TNS-Cu meshes exhibit robust superhydrophilicity and underwater oleophobicity (high oil intrusion pressure) as well as excellent chemical and thermal stability (≈250 °C). They have demonstrated high separation efficiency (oil residue in the filtrate ≤21.3 ppm), remarkable filtration flux (≥400 kL h^−1 ^m^−2^), and sunlight-sensitive anti-fouling properties. Both our theoretical analysis and experimental characterization have confirmed the enhanced light absorption property of TNS-Cu meshes in the visible region (40% of the solar spectrum) and consequently strong anti-fouling capability upon direct solar light illumination. With these features, the proposed approach promises great potential in treating produced oily wastewater from industry and daily life.

For a sustainable environment, nanomaterials with tailored wettability and optical properties can provide innovative and energy-efficient water treatment solutions. Solar energy can be used to treat produced wastewater from industry and daily life with evaporative and membrane filtration approaches^1^,^2^. In the last decade, increased oily wastewater due to rapid industrialization in global cities and multiple oil spill disasters within the marine ecosystems have highlighted the challenges of effective oil/water separation^2^. Clean-up and recovery from an oil spill is difficult and depends upon many factors, including the type of spilled oil, the temperature of the water (affecting evaporation and biodegradation), and the types of shorelines and beaches involved^3^,^4^. Conventional methods for cleaning up such as, bioremediation, controlled burning, dispersants, air flotation, skimming, oil-absorbing materials, and flocculation are limited by low separation efficiency, energy-cost, and complex separation instruments^5^,^6^,^7^. To recover water on a large scale, the oily wastewater treatment membrane should have the capability of high filtration flux, high efficiency, low cost, low energy consumption, and low fouling. In addition, its fabrication process should be suitable for mass production. Table S1 gives a comparison of different oily wastewater treatment methods^8^.

By considering oil and water interface effect towards the selected wettable surface, the design of an oil/water separation process is considered to be important. In the last decade, superhydrophobic/superoleophilic materials in combination with surface chemistry and roughness have been broadly investigated and used to remove oils from water^9^,^10^,^11^. Water being denser than oils tends to form a barrier layer to prevent oil permeation and creates fouling problems^12^. Consequently, superhydrophobic materials are unsuitable for the separation of water rich oil/water mixtures^13^,^14^,^15^,^16^. Inspired from the unique property of fish scales that enable the fish to survive in the oil-polluted waters, recent findings have revealed that a superhydrophilic surface shows underwater oleophobic or even superoleophobic property^17^. Both organic and inorganic superhydrophilic and underwater superoleophobic meshes were fabricated and applied in separating oil/water mixture experiments^17^,^18^,^19^,^20^,^21^.

A series of superwetting materials along with polymer based filtration membranes were employed for the treatment of oily wastewater, driven by external pressure^22^,^23^,^24^,^25^,^26^. But the most serious limitation of these types of membranes is the low flux and quick decline of permeation due to oil adsorption and/or pore plugging by the oil droplets, which lead to severe fouling and difficulty in cleaning^13^,^27^,^28^,^29^,^30^. In addition, their poor thermal stability makes them less applicable to high temperature applications. Inorganic superhydrophilic and underwater superoleophobic meshes, by contrast, were found to have better performance in terms of filtration flux^20^. Therefore, inorganic superhydrophilic and underwater superoleophobic meshes with comparable oil separation efficiency have the potential of meeting the requirement of various industrial applications.

The potential of using inorganic meshes such as stainless steel, and copper meshes for oily wastewater treatment have been investigated^31^. Zhang *et al.* recently reported a superhydrophilic nanowire-haired copper hydroxide mesh with high flux and low oil residue^20^. However, due to presence of sufficient hydroxide groups and poor mechanical stability of nanowires, the mesh surface suffers from fouling problems and low separation efficiency, respectively. Titanium dioxide (TiO_2_), one of the most widely used photocatalyst, has been applied in the fields such as the air and water purification, and self-cleaning. Coating a thin layer of TiO_2_ on those inorganic membranes to achieve anti-fouling property may provide a promising solution. Gao *et al.* reported superoleophilic octadecylphosphonic acid coated TiO_2_ based copper mesh for oil/water separation, followed by methylene blue (MB) degradation while utilizing UV light^21^. The unique oxidation capability is characterized by a common chemical action, which basically relies on the primary reactivity of hydroxal radicals in driving oxidation processes, ultimately resulting in the mineralization of a variety of environmental pollutants^32^,^33^,^34^. However, it is not well understood why nanostructured TiO_2_ and copper are able to achieve better anti-fouling/self-cleaning performance after absorbing both UV and visible light.

In this contribution, we report a robust oily water treatment approach based on superhydrophilic/underwater oleophobic nanostructured titanium dioxide coated copper meshes (TNS-Cu) with high thermal and mechanical stability (Fig. 1). The sunlight absorptivity of the as-fabricated TNS-Cu mesh induces anti-fouling properties. With these TiO_2_ coated meshes, remarkable oil/water separation performance (with a maximum of 401101 L h^−1 ^m^−2^ flux and minimum oil residue of 21.3 ppm) has been attained, without a decline in the flux during continuous use. By utilizing visible light absorption of these TNS-Cu meshes, anti-fouling property on the mesh surface can be achieved to degrade methylene blue (MB) under simulated and direct solar illumination (DSI). Therefore, this solar-assisted anti-fouling TNS-Cu mesh-based technology provides a sustainable, reliable, and energy-efficient way of oily water treatment.

## Results and Discussion

Owing to different interfacial properties of oil and water, the design of mesh requires dual wetting behavior (robust superhydrophilicity and underwater superoleophobicity) for efficient oil/water separation. In practice, the copper mesh was chemically oxidized using NaClO_2_, NaOH, and Na_3_PO_4_·12H_2_O to fabricate nanostructured copper oxide (CuO). SEM image of the nanostructured CuO mesh is depicted in Fig. 2a. The mesh is covered thickly and irregularly by copper oxide nanoflakes with an average length of 800 to 1000 nm, that grow vertically along the mesh walls and intertwine each other. A copper hydroxide nanowire-haired membrane with high oil/water separation efficiency was reported by Zhang *et al.*^20^. However, as mentioned by Yannick *et al.* the copper hydroxide can be transferred to copper oxide at a temperature of 60 °C^35^. The as-fabricated nanostructured copper oxide maintain their nanoflake like morphology even after 15 min of ultrasonic treatment, while copper hydroxide loses nanowire like morphology (Figs S1, S2). It is believed that the CuO phase is stabilized until 300 °C. And around 250 °C the phase transformation of CuO to Cu_2_O starts. As a result, nanoflake like morphology changes into nanograins. The reason is due to the nanoscale effect, which commonly decreases the melting temperature and phase transformation temperature, as frequently reported in other nanomaterials^36^. The predictable reason is the exfoliation that occurs at the interface between the copper substrate and oxidized surface layer. This is due to the thermal expansion difference between the CuO nanowires and copper substrate. Thus beyond 250 °C, the as-fabricated CuO would easily be cracked and peeled off from substrates^37^. The XRD pattern has confirmed the existence of the crystal of CuO and Cu (Fig. 2b), which gives a single-phase with a monoclinic structure. The intensities and positions of the peaks are in good agreement with the reported values (JCPDS file No. 05–661). The two extremely strong peaks correspond to the (111) and (200) crystal planes of the Cu mesh. No peaks of impurities are found in XRD pattern. The peaks are broad due to the nano-size effect.

To induce the anti-fouling and self-cleaning properties, nanostructured TiO_2_ was coated on Cu and CuO mesh (TNS-CuO). A semiconductor on the surface of metal could exhibit certain interesting phenomena, like self-cleaning and dye degradation via light absorption^38^. The details of fabrication methods of depositing nanostructured TiO_2_ on the mesh surface are provided in supplementary information. Figure 2c,d presents the SEM images of as-fabricated TNS-CuO-I and TNS-Cu-II. The mesh surface shows the nanoparticle coagulated-like morphology after TiO_2_ deposition using LBL approach on nanostructured covered copper mesh. As shown in Fig. 2d, TNS-Cu-II, which was prepared with a sputter-hydrothermal coating method, were found with nanowire-like morphology on its surface. The diameter and length of the nanowire are around 10 and 100 nm, respectively. SEM images for TNS-CuO-I and TNS-Cu-II and are provided in supporting information. To validate the existence of TiO_2_, X-ray photoelectron spectroscopy (XPS) results for TNS-CuO-I and TNS-Cu-II are shown in Fig. 2e,f. The peak situated at *E*_*b*_ (Ti 2p3/2) = 459.08 eV was assigned to Ti^4+^, which indicates the existence of Ti(IV) Oxide. The peak at 933.85 eV represents the existence of pure Cu (II) oxide^39^. The O1s oxygen peak at 531.08 eV represents the binding energy Ti-O bond (529.6–530.2 eV) and Cu-O bond (529.6 eV). The peaks of sodium, carbon, and calcium in Fig. S3 are as a result of contamination. However, the presence of chloride was the result of adding of hydrochloride acid as a catalyst in the sample preparation process. Our EDS results also confirmed the presence of titanium on TNS-Cu and TNS-CuO meshes, as shown in supporting information. Due to the presence of copper oxide in the TNS-CuO-I mesh, the oxygen content is higher than that in TNS-Cu mesh. The existence of chloride element is the result of using hydrochloric acid as a catalyst during the TiO_2_ coating. EDS results of Sample TNS-Cu-II and Sample TNS-Cu-III are presented in supporting information, confirming the presence of titanium. The content of titanium is 6.4 wt%, while on the right is 0.06% (wt%). Copper meshes used here were made of phosphor bronze instead of pure copper, because it’s difficult to fabricate copper mesh with such low pore size. This explains the existence of 1 wt% tin element.

To check the wetting properties, nanostructured copper oxide and TNS-Cu meshes were characterized using contact angle goniometer. The TNS-Cu substrates displayed roughness in nano to microscale level which is crucial for controlling the surface wettability (AFM image shown in the supporting information)^40^. The water contact angle (WCA) in the air and underwater oil contact angle (OCA) of these meshes are presented in Fig. 3a. The measured values for WCAs of nanostructured CuO, TNS-Cu-I, TNS-Cu-II, and TNS-Cu-III, are 28.6, 24.3, 18.2, and 0°, while the corresponding underwater OCAs are 159, 144.75, 155, and 139.6°, respectively. These values confirm the superhydrophilic and underwater superoleophobic property of as-fabricated meshes. The reported values of WCA were measured at 600 ms after the droplet was placed on the mesh surface. The superhydrophilic and underwater superoleophobic property is attributed to the roughness, which is associated with nanostructures growing on micro wires, and the hydrophilic nature of TiO_2_ and nanostructured copper. When this TNS-Cu mesh is immersed in water, water could be trapped within irregular nanostructures to form a complex oil/water/solid interface in the presence of oil. During the oil/water separation process, a series of immiscible oil/water mixtures were poured onto the meshes with different pore sizes individually. Water immediately permeated through the meshes and oils were retained above, see set-up in supporting information. No external driving force was imposed during the fast separation process (within 100 sec). The residual oil content in the filtered water was measured after only one separation cycle. As shown in Fig. 3b, for all types of TNS Cu meshes, the measured oil content in the filtrate of the oil/water mixture was low. The oil residue in the filtrate using TNS Cu of mesh number 120, 200, 300, and 500 were 157, 38.5, 34.4, and 31.8 ppm, respectively. With smaller pore size, the lowest oil residue is achieved. For confirmation, we also measured the total organic carbon (TOC) value in the permeate after oil/water mixture separation. Compared to the reported results, this oily wastewater treatment process has a much higher separation efficiency (>99.99%) and ultra-low chemical oxygen demand (COD) 31.8 ± 4.8 ppm and TOC 10.0 ± 3.5 ppm values in the filtrate. The water fluxes of all the meshes were also calculated by measuring the time for oil/water mixture of a certain volume passing through the specific area of the NS copper mesh. The measured average fluxes for TNS-Cu of mesh number 120, 200, 300, and 500 were 895248, 554036, 472215, and 381240 L h^−1 ^m^−2^. As expected, the flux and the oil residue reduce with the increase of mesh number, or equivalently the decrease of pore size. Similarly, the measured oil residues for TNS-Cu-I, TNS-Cu-II, and TNS-Cu-III meshes with same pore size (32 μm, M500) are 21.3, 10.8, and 44.5 ppm, respectively. And their corresponding fluxes are 401100, 350535, and 342628 L h^−1 ^m^−2^, respectively. With only 21.3 ppm oil residue and 401101 L h^−1 ^m^−2^ flux, TiO_2_ coated meshes have shown remarkable performance for treatment of oily wastewater.

The systematic design of membranes for oil/water separation requires the parameterization of two important physical characteristics, breakthrough height and intrusion pressure, to quantify the maximum hydrostatic pressure that a membrane could sustain. Before separating on the mesh should be wet so that water could be trapped within the nanoflakes on the mesh wire surface. This adsorbed water layer plays two distinct roles in oil/water separation: first, it prevents oil droplets from contacting with the mesh in the separation process; second, this layer provides a pathway for the water droplets from the oil/water mixture to permeate to the opposite side of the coated mesh. As the matter of fact, we develop a simple analytical model to predict the contact line of oil, water, and solid interfaces in the TNS-Cu mesh. As shown in Fig. 3c, the grey, blue, and yellow parts represent the solid, water, and oil phases, respectively. Throughout the separation process, water permeates through the mesh, leaving oil on the top side. When concentration of oil increases, the oil height (*H*) and hydrostatic pressure increases, and the oil gradually started to move into the pores along the mesh pore wall surface with the same apparent contact angle. Breakthrough takes place when the curvature of the oil/water meniscus reaches to the minimum, which happens when the tangent line of the oil/water interface at the triple-phase point is perpendicular to a horizontal line. Since the pores of the meshes are in square shape as seen from the top, the effective pore size cannot be just considered as the actual pore width *p*. Instead, an average of the actual pore width and diagonal of the pore should be taken into account:


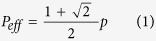


Another point needs to be considered: only the fraction of the oil/water surface tension along the tangent line of the oil/solid surface at the triple-phase point plays a critical role in holding the oil. In order to quantify the breakthrough height and intrusion pressure of meshes, we propose a theoretical model as below,


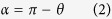



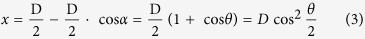



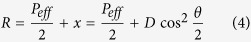



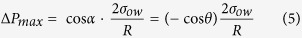



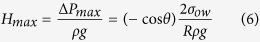


where, *θ* is the oil contact angle in water; *D* is the diameter of the wire; *R* is the radius of the circular surface; *p* is the width of the gap; *P*_*eff*_ is the effective pore size: Δ*P*_*max*_ is the oil breakthrough/intrusion pressure; *g* is the gravity of the per square meter of oil; *σ* is the interface force of oil and water; *ρ* is the density of oil; *H*_*max*_ is the maximum height of the retained oil; *α* is the angle as shown in the Fig. 3c. We can substitute the effective pore size (as the pore shape is square) in Eq. (4) to evaluate the effective radius of mesh wire and predict the oil breakthrough/intrusion pressure as in Eq. (5). It also implies that over the critical height, *H*_*max*_, the oil starts flowing downward and penetrating the TNS-Cu mesh. According to equation (6), with the example of soybean oil which has a surface tension of 27.6 dyne/cm, the intrusion pressure – pore size curve was plotted, as shown in Fig. 3b. According to this model, when the underwater OCA is fixed at 159°, the breakthrough heights of TNS-Cu meshes with a pore size of 32, 46, 74, and 110 μm are 0.67, 0.47, 0.29, and 0.19 m, respectively. And their corresponding intrusion pressures are 4.3, 3.0, 1.9, and 1.3 kPa, respectively. The experimentally measured *H*_*max*_ values for each membrane with a pore size of 32, 46, 74, and 110 μm are 0.68, 0.45, 0.30, and 0.21 m, respectively. These experimental results were also used to obtain the intrusion pressures using equation (6), as shown in Fig. 3d, which are in good agreement with analytical modelling results. This analysis indicates the capability of the TNS-Cu meshes of treating a large amount of an oil/water mixture for long-term usage.

To test the stability and antifouling property of the TNS-Cu meshes, continuous separation of oil/water mixtures was performed. At different time intervals, flux and separation efficiency of filtered water were measured (detailed is provided in supporting information). During continuous oil/water separation for seven days, water continuously permeates through the membrane and simultaneously oil accumulated above the membrane was cross-flew away to avoid blocking in water permeation. During the whole testing process, no obvious decrease in flux occurred and the oil content in the filtrate remains around 25 ppm, as shown in Fig. 4a. The change in wettability properties of mesh was also monitored at different time intervals, and unnoticeable change in WCA and underwater OCA have been observed (Fig. 4b). This result indicates the capability of the membrane for treating a large amount of an oil/water mixture without altering in flux and separation capability.

To investigate the anti-fouling property, TNS-Cu substrate was dipped in 500 ppm MB solution for 2 hours in the dark. The organics (MB) adsorption within the nanostructures of the sample altered the intrinsic superhydrophilicity by changing the WCA from 0 to 56 ± 3°. To restore the Superhydrophilicity, we illuminated sample surface under 1 sun solar simulator and DSI on roof top. The average measured DSI during the measurement time was 416 ± 3 W/m^2^. After 30 min of irradiation, the sample restored its superhydrophilicity, which is a clear indication of MB degradation adsorbed within the nanostructures of sample solely using sunlight. The TNS-Cu sample has shown an excellent reproducibility for continuous seven days experiment with the same sample (Fig. 4c). To verify the role of nanostructured TiO_2_ coated mesh, solution based MB degradation experiments were also performed using 1 SUN intensity irradiation. After 2 h of illumination, the resulted solution was characterized by UV/VIS/NIR spectrometer. The results are shown in supporting information. The decrease of absorptance peak is the result of photocatalytic decomposition of MB by TiO_2_ under illumination. The spectral absorptance of MB(aq) degradation confirms the photocatalytic properties of those TNS-Cu and TNS-CuO meshes under 1 SUN irradiation and their anti-fouling properties. For comparison, the experiments were also performed at rooftop by using DSI. The corresponding results are shown in supporting information. The dash and dotted lines present the MB decomposition when the samples were irradiated with side walls covered and uncovered, see details of setup in supporting information. The extent of photocatalytic degradation could be evaluated by measuring the absorbance of the solution at 664 nm. The degradation of efficiency of MB was calculated using the equation:


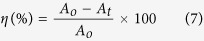


where *A*_*o*_ present the initial absorbance, and *A*_*t*_ represents the absorbance after time *t* min reaction of the MB at the characteristic absorption wavelength of 664 nm. The results of MB degradation efficiency are summarized in Table 1. The thin film of TiO_2_ of thickness of 120 nm on glass and copper has shown MB degradation efficiency of 25 and 33%, respectively. This difference of efficiency is attributed to the visible light absorption by the copper substrate to facilitate charge transfer from metal to semiconductor, and also to inhibit electron-hole pair recombination in semiconductor. All the samples of TNS-Cu have shown MB degradation efficiency between 43.77 to 51.98% using solar simulator as an irradiation source. In case of DSI using TNS-Cu-III, the degradation efficiency improved from 47.86 to 52.14 and 64.92% under the measured value of solar flux of 416 ± 3. The nanostructure morphology has enhanced the degradation efficiency due to increase in surface area.

To quantify the role of sunlight absorption capabilities of nanostructured TiO_2_ coated Cu mesh, we compared an absorptance spectra of thin film of 120 nm thick TiO_2_ on glass and Cu substrate. Since the glass is transparent, the absorptance of TiO_2_ on glass substrate is equivalent to the absorptance of a single layer of TiO_2_. Figure 5a showed the absorbance spectrum of TiO_2_ on glass, which has presented an absorbance peak before 400 nm. The flat region between 400–800 nm demonstrated that TiO_2_ itself has not absorbed any light from the visible range. The inset graph of Fig. 5a presented the absorbance spectrum of 120 nm thick TiO_2_ on Cu substrate. Besides absorbance in the UV region, a strong absorbance peak is also observed in the visible region. Thus TiO_2_ coated on Cu can absorb the light from UV as well as visible region of solar spectrum, thus provided a strong evidence for solar-assisted anti-fouling properties. Optical absorption measurements are widely used to understand the any change in the band gap, valence band tails and excited state lifetime of thin films^41^,^42^. Several models are used to determine the optical properties of semiconductors. The most widespread method is the Tauc model which allows us to derive the band gap energy Eg from E(ε)^n^ as a function of the incident energy E. The Tauc optical band gap associated with the thin films is determined through an extrapolation of the linear trend observed in the spectral dependence of (α*h*ν)^n^ over a limited range of photon energies *h*ν^42^. Figure 5b indicates that TiO_2_ thin films on glass have only one strong absorption band around 3.65 eV, while TiO_2_ thin films on copper has two strong absorption band at 3.15 and 1.80 eV. In the later case, the first adsorption around 3.15 eV is attributed to the direct transition within the TiO_2_ while second adsorption around 1.80 eV corresponds to the transition within the copper. The unique combination of TNS-Cu has even reduced the band gap from 3.65 to 3.15 eV. The Tauc adsorption curves provide a strong evidence of absorption of solar light in the visible region with alteration of the band gap of TNS-Cu films.

In order to theoretically investigate the optical properties of our samples, we used the transfer matrix approach^43^ to calculate the absorption spectra for three different stacks, where the refractive indexes of these materials were taken from Palik *et al.*^44^. For the simplicity of theoretical analysis, nanoscale roughness of these samples were neglected. The stacks include TiO_2_/glass, TiO_2_/Cu, and TiO_2_/CuO/Cu. The thicknesses of TiO_2_ and CuO were chosen as 100 and 500 nm, respectively. In Fig. 6a, our theoretical calculation results show that the absorption of TiO_2_ on glass substrate is only in the UV region, which is in agreement with the experimental results shown in Fig. 6b. The TiO_2_/Cu stacked structure shows a strong absorption band in the visible range due to the absorption of metallic Cu substrate. Lastly, the absorption spectrum of TiO_2_/CuO/Cu possesses a wide absorption broadband over the full visible range. The absorption at longer wavelength is due to the intrinsic absorption of CuO in the visible range^45^, which is also confirmed by our theoretical results. Figure 6b displayed the measured absorptance spectra of the as-prepared TNS-Cu-I and TNS-CuO-II substrates. In both samples, absorption was found during the visible light range. The absorptance spectrum of TNS-Cu-I substrate started to decrease abruptly at 550 nm, while TNS-CuO-I substrate has the best absorption performance in the full visible range. The wide and flat absorption during the visible range is due to the presence of nanostructures and the presence of CuO between Cu and TiO_2_. Currently, it is difficult to claim that the presence of CuO is helpful in our photocatalytic performance. Nevertheless, the strong absorption in the visible range by TNS-Cu substrate has provided an evidence of visible light absorption and enhanced MB degradation with the broadband solar spectrum of direct incident sunlight. Therefore, nanostructured TiO_2_ coated copper meshes are able to achieve both effective oil/water separation and solar-assisted organic degradation. TiO_2_ coated on copper mesh using hydrothermal approach has the advantages of low fabrication cost, better oil/water separation performance, and higher photodegradation efficiency using solar light.

## Conclusions

In summary, we successfully fabricated sunlight-sensitive anti-fouling superhydrophilic/underwater oleophobic nanostructured TNS-Cu meshes with the oil contact angle more than 159°. By selecting the appropriate etching conditions and pore size, the best nanostructured mesh itself demonstrated a very high filtration flux (>400 kL h^−1 ^m^−2^), ultra-low oil residue in the permeate (25 ppm), long water retention time (more than 1 h natural evaporation to air) and solar-assisted cleaning. Both our analytical model prediction and experimental results have shown the desired oil intrusion pressure and breakthrough height can be obtained by simply changing the pore size. For instance, a TNS-Cu mesh with 32 μm pore size can sustain high oil intrusion pressure (4.3 kPa) with a breakthrough height of 0.67 m. The continuous oil/water separation experiments have proven the durability of TNS-Cu meshes for long term use. The enhanced light absorption property of TNS-Cu meshes in the visible region (40% of the solar spectrum) has enabled them to obtain satisfactory anti-fouling performance upon direct solar light illumination. Furthermore, our optical modeling and experimental analyses suggest that these TNS-Cu meshes exhibit strong absorption in a wide UV-visible light range, which favors the anti-fouling and organic degradation. The proposed scalable and low-cost approach is a valuable contribution to the development of large-scale solar-assisted water treatment technologies.

## Methods

### Preparation of nanostructured meshes

**Figure 1:**
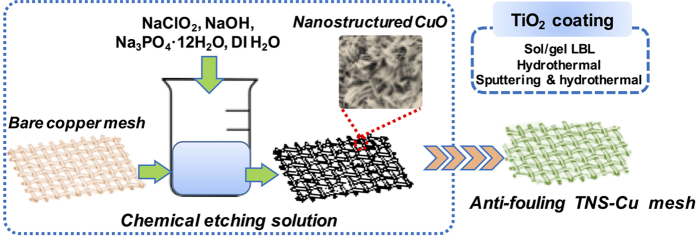
Schematic diagram of fabrication of nanostructured TNS-Cu mesh.

**Figure 2:**
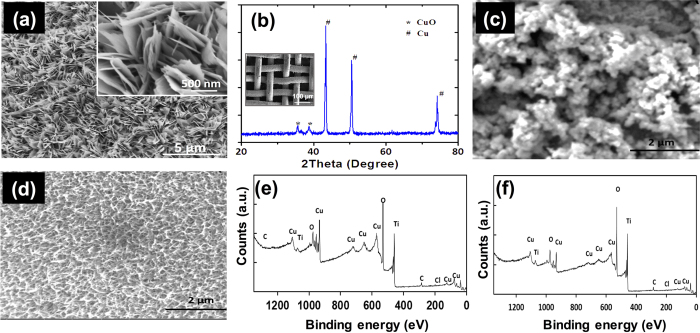
Characterization of nanostructured CuO and TNS-Cu meshes. (**a**) SEM images of nanostructured CuO mesh after chemical oxidation using 5:10:3.75 proportion of NaOH/Na_3_PO_4_/NaClO_2_. (**b**) XRD pattern of nanostructured CuO mesh, which demonstrates that the components of nanostructured mesh are CuO and Cu, inset shows SEM image of nanostructured copper mesh. (**c**,**d**) SEM image of TNS-CuO-I and TNS-Cu-II with LBL assembly method and sputter-hydrothermal method, respectively. (**e**,**f**) XPS results of (**c**,**d**).

**Figure 3:**
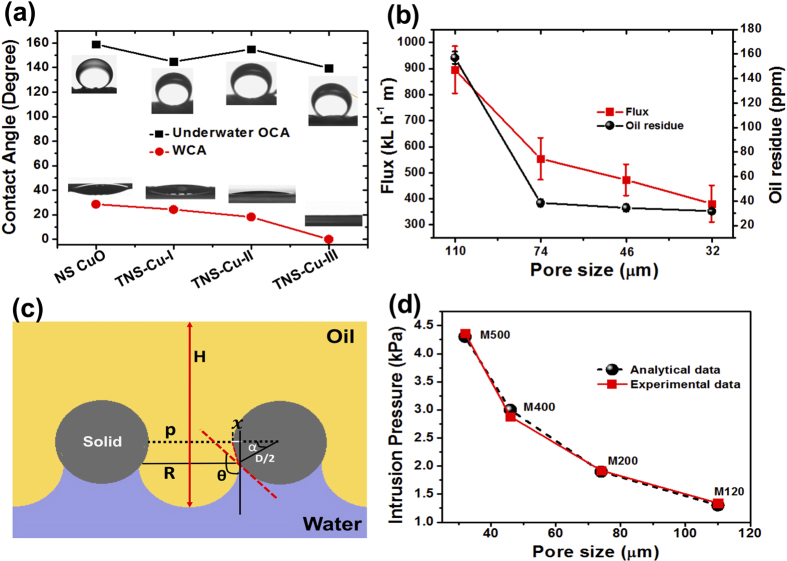
Wettability and oil/water separation performance of nanostructured meshes. (**a**) WCA and underwater OCA and the respective optical snapshot of liquid droplet on various meshes. (**b**) Flux and oil residue (COD measurement) in the permeate measured after oil/water separation using nanostructured membranes with various pore size. The reported values were obtained after first filtration. (**c**) Schematics of oil on water wetted TNS Cu mesh. (**d**) Predicted and measured intrusion pressures of TNS Cu mesh with different pore size.

**Figure 4:**
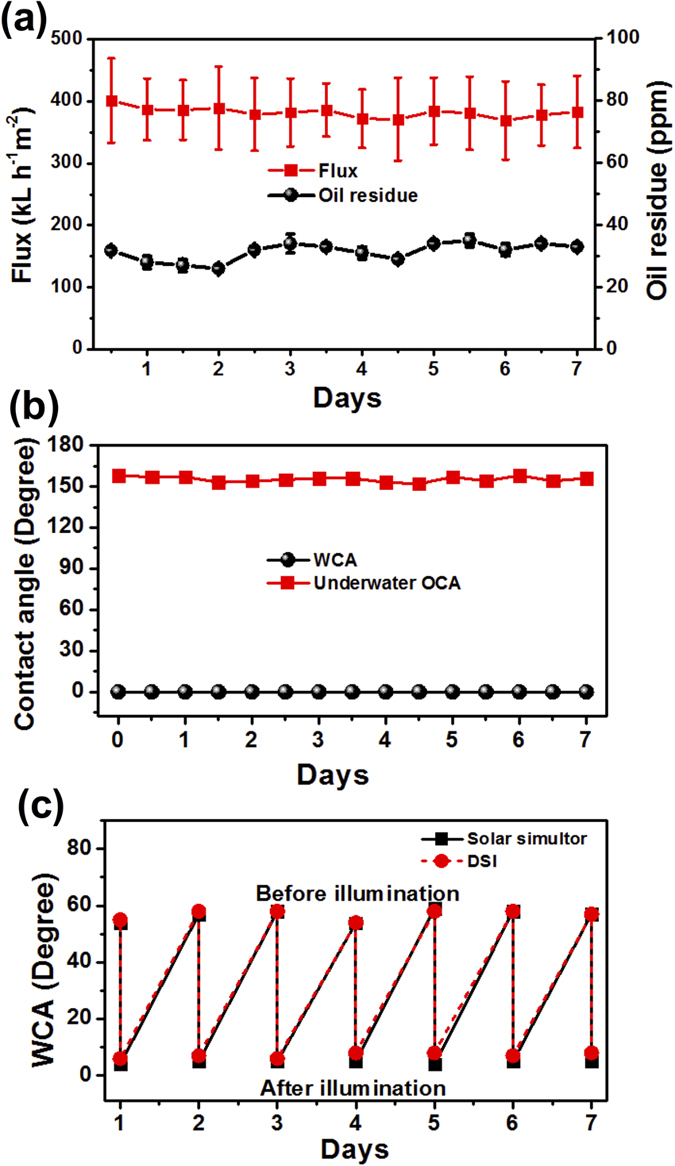
Durability and anti-fouling performance of nanostructured meshes. (**a**) Change in flux and oil residues with time. (**b**) Change in WCA and underwater OCA with time. (**c**) WCA dependence of TNS-Cu substrate before and after illumination with 1sun solar simulator and DSI on roof top. The samples were dipped in 500 ppm solution of MB(aq).

**Figure 5:**
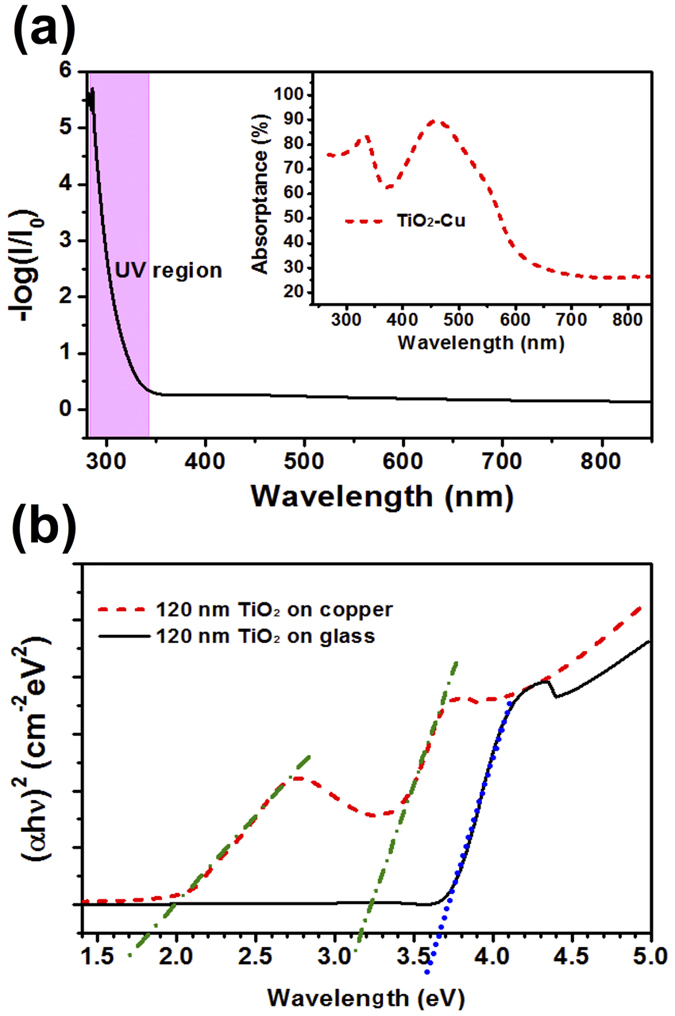
UV-Vis characterization and Tauc plot for band gap measurement. (**a**) The measured absorptance spectrum of the thin films of 120 nm thick TiO_2_ coated on glass and Cu substrate (inset). (**b**) Plot of (α*h*ν)^2^ versus photon energy *h*ν for thin films of TiO_2_ on glass and copper substrates.

**Figure 6:**
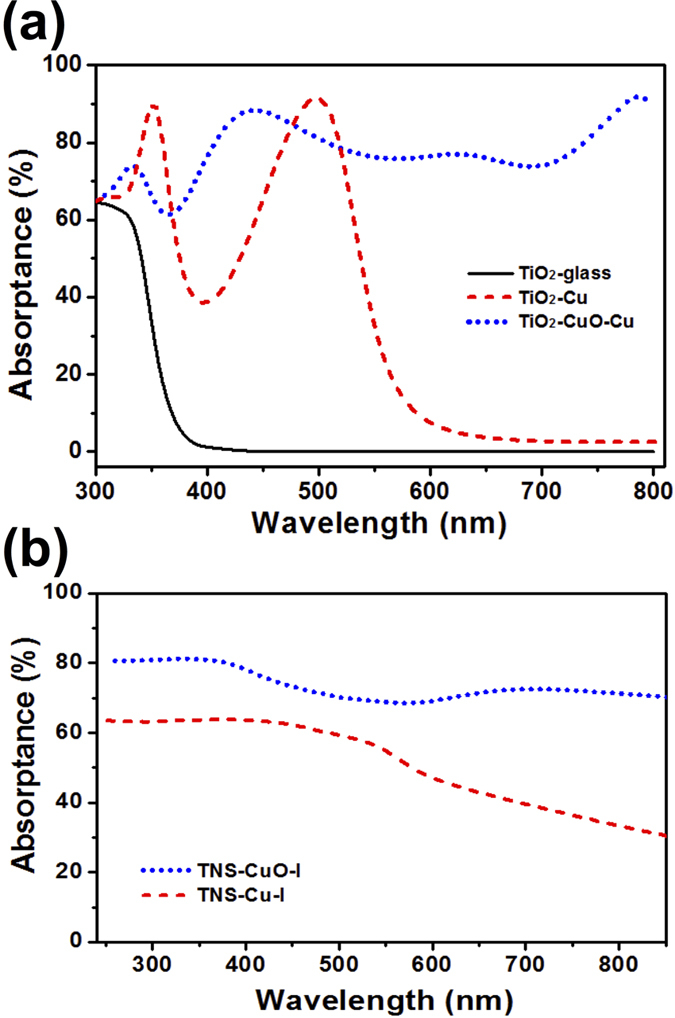
Simulated and experimental light absorption characteristics. (**a**) The predicted absorptance spectra of thin films of 120 nm thick TiO_2_ on glass, Cu, and 500 nm thick CuO/Cu substrates. (**b**) The measured absorptance spectra of TNS-Cu-I and TNS-CuO-I substrates.

Pre-cleaned copper sheet and meshes (M120, M200, M300, and M500, pore size 110, 74, 46, and 32 μm, Anpin Keian Metal Meshes, Co. LTD) were immersed in aqueous solutions of NaClO_2_, NaOH, and Na_3_PO_4_·12H_2_O (Sigma Aldrich) with different mole ratios for 5 min at 90 °C. Details of chemical oxidation using various molar ratios of oxidants is given in supporting information. After chemical oxidation, the intrinsic reddish brown color of copper meshes turned to deep black. TiO_2_ was coated on nanostructured CuO sheet/mesh using various approaches, like sol gel layer by layer (LBL) assembly, hydrothermal, sputtering followed by hydrothermal method. As-deposited TiO_2_ nanostructures on Cu and CuO substrates using various approaches were named as TNS/Cu-m. Where, ‘m’ corresponds to “I, II, and III” for LBL assembly, sputtering followed by hydrothermal methods, and hydrothermal treatment, respectively. Thin film of TiO_2_ 120 nm thickness was deposited on glass and Cu substrate using sputtering of 99.9% TiO_2_ target. The schematic of fabrication of TNS- Cu mesh is given in Fig. 1.

### Oil/Water separation and anti-fouling experiments

Oil/water mixture was prepared by mixing water and sunflower oil (SO) with the volume ratio 9:1 v/v for separation experiments. For convenience, oil was dyed red using oil red (Nanjing Aodo Funi Technology Co. LTD). The detail is provided in supporting information. Diesel oil (DO) and crude oil (CO) samples were provided by a local oil company in Abu Dhabi. The residual oil content in the filtrate was calculated by measuring chemical oxygen demand (COD) and total organic carbon (TOC) values. For anti-fouling studies, various TNS-Cu samples were immersed in 20 mL of as-prepared 200 ppm MB (Sigma Aldrich) solution in a 100 mL beaker. The distance between the sample and the light source was 5 cm. Then samples were illuminated under a solar simulator with 1 SUN irradiation density. A few experiments were also run directly under sunlight at rooftop. The solar illumination setup is shown in supporting information.

### Instruments and Characterization

The morphology and energy-dispersive X-ray spectroscopic (EDS) measurements of mesh was examined by a scanning electron microscope (SEM) (Nova NanoSEM 650 FEI). Small-angle X-ray diffraction (XRD) pattern was examined using X’Pert Pro MPD XRD spectrometer. Water contact angle (WCA) (3 μL) and oil contact angle (OCA) (3 μL) measurements were performed using contact angle goniometer (DM-501, Kyowa Interface Science Co. Ltd). COD and TOC measurements were performed by using DR2800 COD spectrophotometer and Vario TOC cube, respectively. Absorbance spectrums (from 250–800 nm) were recorded using LAMBDA 1050 UV/VIS/NIR Spectrophotometer. The XPS analyzes were performed using the K-Alpha XPS at the CNS at Harvard Cypher and AC160TS.

## Additional Information

**How to cite this article**: Liu, H. R. *et al.* Sunlight-Sensitive Anti-Fouling Nanostructured TiO_2_ coated Cu Meshes for Ultrafast Oily Water Treatment. *Sci. Rep.* **6**, 25414; doi: 10.1038/srep25414 (2016).

## Supplementary Material

Supporting Information

## Figures and Tables

**Table 1:** Degradation efficiency of MB in solution using thin films of TiO_2_ (120 nm) on glass and Cu substrate and TNS-Cu-m meshes illuminated for 2 h.

Sample name	Irradiation source	*η* (%)
TiO_2_*-glass	Solar simulator	25.22
TiO_2_*-Cu	Solar simulator	33.41
TNS-Cu-I	Solar simulator	51.98
TNS-CuO-I	Solar simulator	50.83
TNS-Cu-II	Solar simulator	43.77
TNS-Cu-III	Solar simulator	47.86
TNS-Cu-III	DSI# (from top)	52.14
TNS-Cu-III	DSI# (from all sides)	64.92

^*^Thickness = 120 nm.

^#^Direct solar irradiation.
