# Supplementary material for: Sunlight-Sensitive Anti-Fouling Nanostructured TiO2 coated Cu Meshes for Ultrafast Oily Water Treatment
Source: Sci Rep. 2016 May 10;6:25414. doi: 10.1038/srep25414 (PMC4861963; doi:10.1038/srep25414)
Supplement: Supporting Information [file srep25414-s1.pdf]

Sunlight-Sensitive Anti-Fouling Nanostructured TiO_2 coated Cu Meshes for Ultrafast Oily Water Treatment

HaoRan Liu[+], Aikifa Raza[+], Abulimiti Aili, JinYou Lu, Amal Al Ghaferi, and TieJun Zhang*

Department of Mechanical and Materials Engineering, Masdar Institute of Science and Technology, P.O. Box 54224, Abu Dhabi, UAE

*tjzhang@masdar.ac.ae

+Both authors have equally contributed to this work

Table S1. Comparison of different oily wastewater pretreatment.

Treatment Method	Produced Water	Before Treatment	After Treatment
MF Membrane Separation	Oilfield	COD= 1600 ppm<br>O&G= 220 ppm	COD= 960 ppm<br>O&G=19 ppm<br>Phenol = 1.9 ppm
Induced-air Floatation	Oilfield	-	COD= 595 ppm<br>TOC=115 ppm
Dissolved Gas Floatation	Oilfield	-	BOD= 180 ppm<br>TDS = 20 g/L

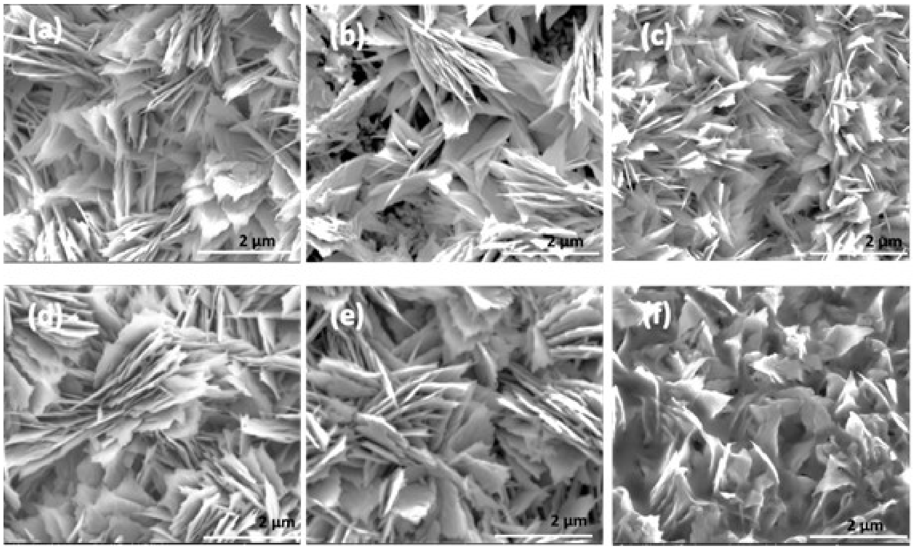

Figure S1. SEM images of as prepared NS Cu-500 mesh after 24 hours annealing treatment at different temperature, (a) is without treatment, (b), (c), (d), (e), and (f) are SEM images after treating at 50, 100, 150, 200, and 250°C, respectively.

In order to test the temperature stability, the as prepared etched copper mesh sample ($NaOH/Na_3PO_4/NaClO_2$ =5:10:3.75) were heat treated at temperature of 50, 100, 150, 200, 250°C for 24 hours. The result SEM images were shown in Figure S1.

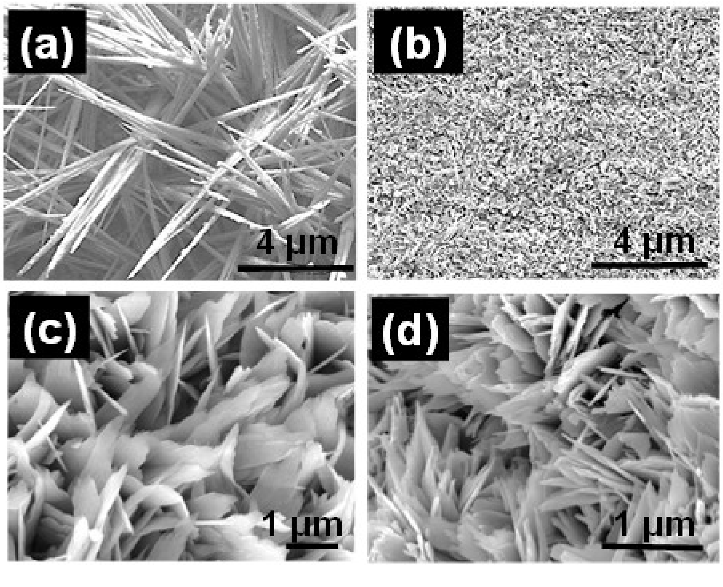

Figure S2. (a-b) Copper hydroxide and (c-d) copper oxide nanostructure before and after ultrasonic cleaning treatment for 15 min.

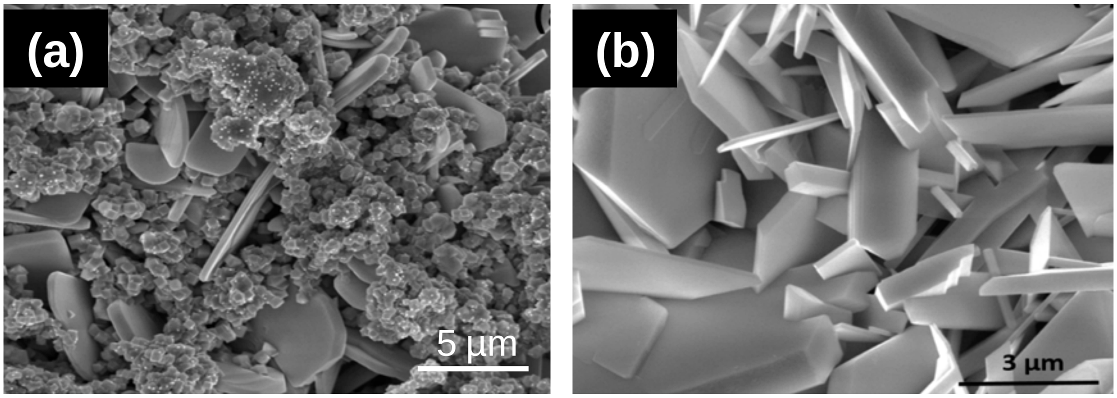

Figure S3. (a,b) SEM images of TNS-Cu-I and TNS-CuO-I meshes.

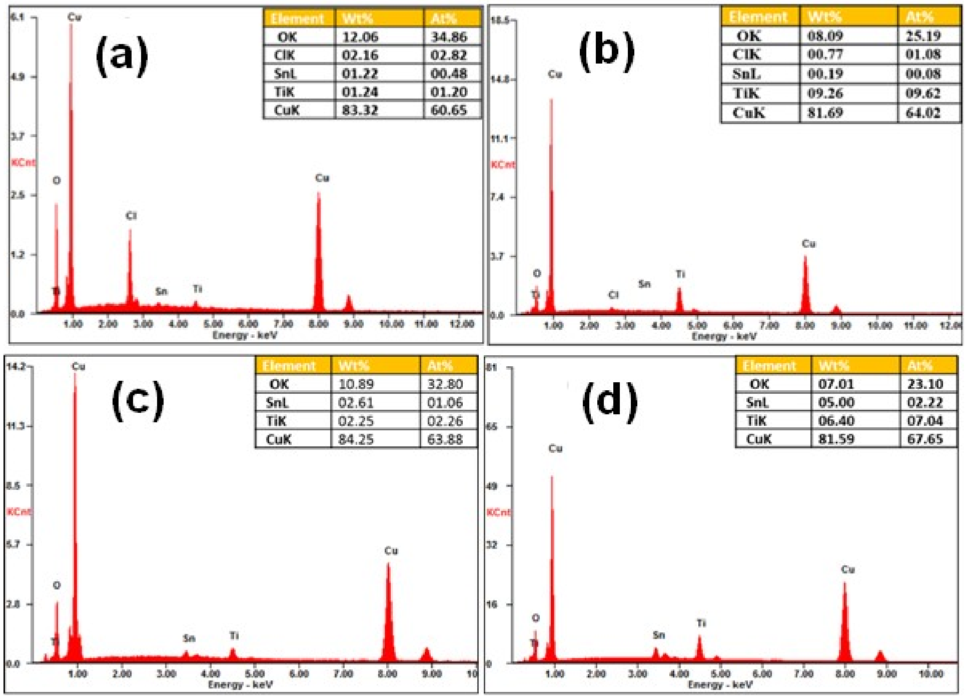

Figure S4. (a-d) EDS spectrograph of TNS-Cu-I, TNS-CuO-I, TNS-Cu-II, and TNS-Cu-III meshes.

Fabrication of TiO_2 coated nanostructured copper meshes (TNS-Cu-m)

Hydrothermal Method (TNS-Cu-I)

Figure S4 illustrated the process of the hydrothermal method. The hydrothermal solution used here was made of 4 mL titanium (IV) n-butoxide, 30 mLIsopropanol, and only one

drop of hydrochloric acid. Target samples and the solution were transferred into the autoclave and then heated to 200°C. After 2 hours, the autoclave reactor was cooled down with oven naturally.

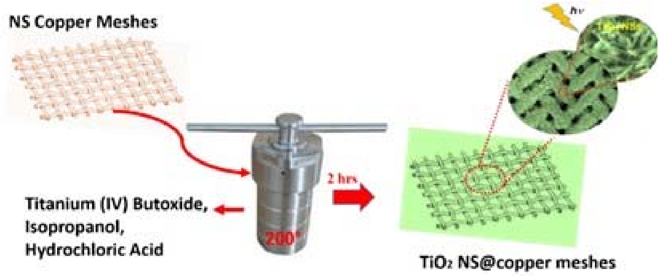

Figure S5. Schematic diagram of titanium dioxide coating by hydrothermal method (TNS-Cu-I and TNS-Cu-II).

Sputtering-hydrothermal Coating Method (TNS-Cu-II)

Depositions were done by using DC Magnetron sputtering (as shown in Figure S5). Titanium target of 99.999% purity was used. In order to get enough thick titanium layer, the condition was set at 100 W power and 60 minutes operation time. At 100 W, the sputtering speed is 0.32 angstrom per second. Therefore, the calculated thickness of titanium is 115.2 nm. Before sputtering target samples were ultrasonic washed with deionized water, acetone, isopropanol alcohol, and deionized water each 6 minutes in order. After sputtering, samples were ultrasonic washed with acetone, isopropanol alcohol and deionized water each 3 minutes in order. After that, all the samples were immersed in DI water to avoid contamination. Then, samples were taken out one by one, rinsed it in high-pressure water and then dried by using high-pressure nitrogen.

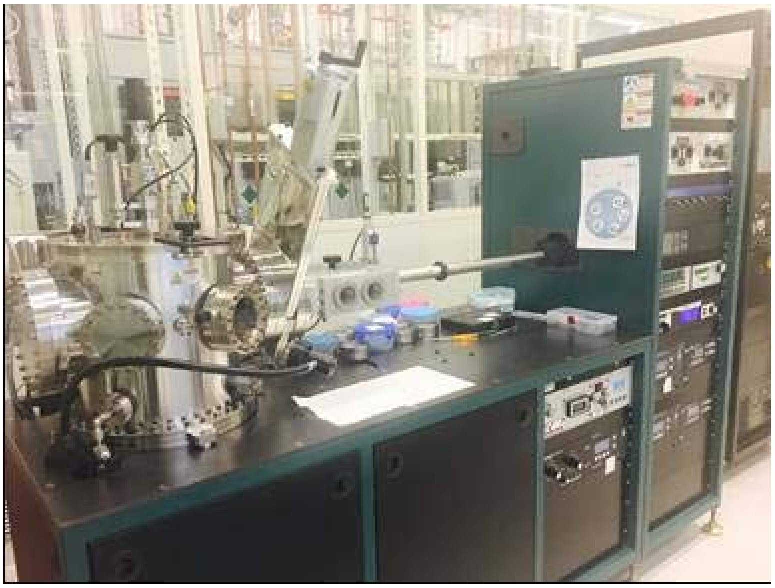

Figure S6. Optical image of AJA magnetron sputter system.

In order to get titanium dioxide and increase the roughness of sample surfaces at the same time, the as-prepared samples were treated with the hydrothermal method after sputter coating. 40 mL of 1 M sodium hydroxide solution was added into the autoclave reactor with the as-prepared samples. Then, they were heated to 230°C and keep at this temperature for 30 minutes. After cooling down, samples were taken out and washed with DI water.

LBL Assembly Method (TNS-Cu-III)

500M copper meshes and nanostructured copper meshes were cleaned in an ultrasonic bath with acetone for 15 minutes and then rinsed with methanol, isopropyl alcohol, and DI water. A clear solution of 0.6 mL $TiOBu_4$ and 20 mL ethanol was prepared. Then, the LBL assembly approach was used to coat titanium dioxide by using the following procedure.

1) The as cleaned copper meshes were immersed into the clear solution for 10 minutes.
2) Samples were taken out, and the water contact angle was measured.
3) The samples were immersed into deionized water for 3 minutes.

4) Samples were taken out, and the water contact angle was measured again, and difference of contact angles was noted.
5) The procedure was repeated from step 1.
6) After several cycles, the prepared samples were annealed at 450°C.

As the Figure S6 described, $TiOBu_4$ will first react with the hydroxyl groups on the surface of the copper mesh. After the reaction, the surface becomes more hydrophobic. Then, hydrolysis reaction makes the surface more hydrophilic.

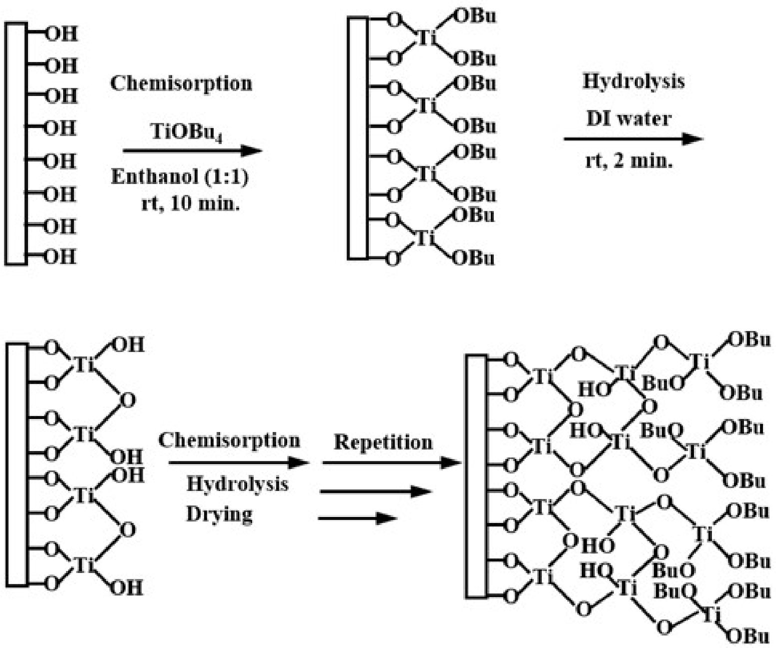

Figure S7. The mechanism of layer by layer coating. Reprinted with permission from [3].

Figure S7 showed the EDS result of samples prepared by LBL. The content of titanium element is 6.4% (on weight) while on the right is 0.06% (w%). Figure S9b and Figure S9c demonstrated the EDS mapping results. As it shown in the Figure S9c, the distribution of oxygen is uniform but titanium is not, which indicates that titanium dioxide did not fully cover the copper mesh.

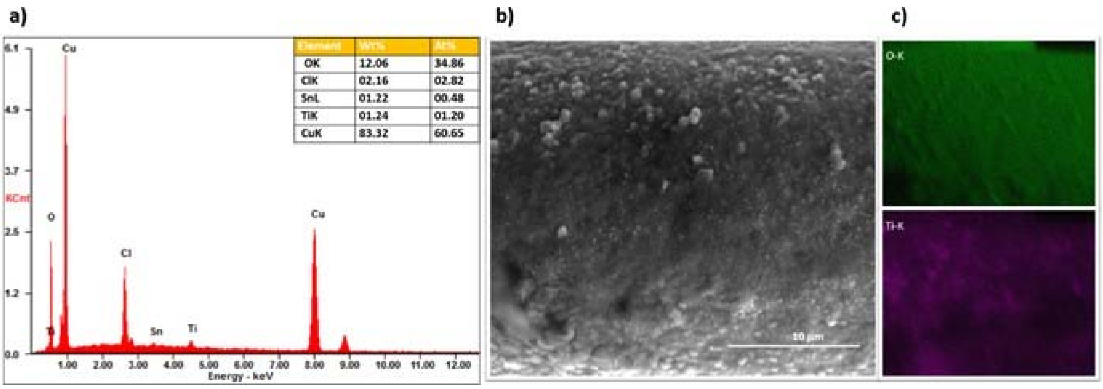

Figure S8. (a) EDS result of titanium dioxide coated on bare copper meshes with LBL method; (b) SEM image, and (c) EDS mapping of oxygen and titanium elements in the corresponding sample, the presence of tin is because of the impurity in copper mesh.

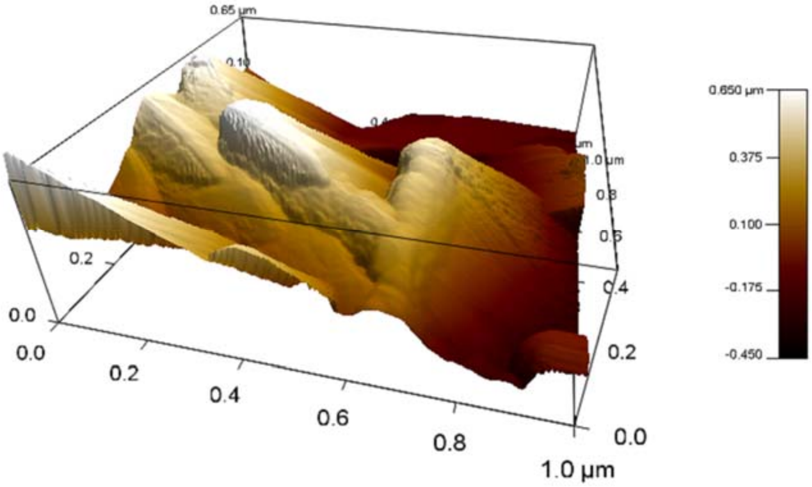

Figure S9. AFM image of TNS-Cu substrate.

Oil/water separation experiments

In the separation experiment, a 150 mL filtration flask was used. The copper mesh was fixed on the top of a 10 mm wide tube. Before the experiment, copper mesh was wetted by DI water. Then, the oil/water mixture was poured into the copper mesh fitted flask from the top and the separation was solely driven by gravity. To mimic the continuous oil/water separation experiments, the mesh was adjusted on to the glass cylinder in a way that the lower part of the mesh was in continuous contact with water and upper part was in contact with oil/water mixture for continuous seven days of experiments. During each day at different time intervals, filtration flux was measured and filtered samples were collected to measure the COD values.

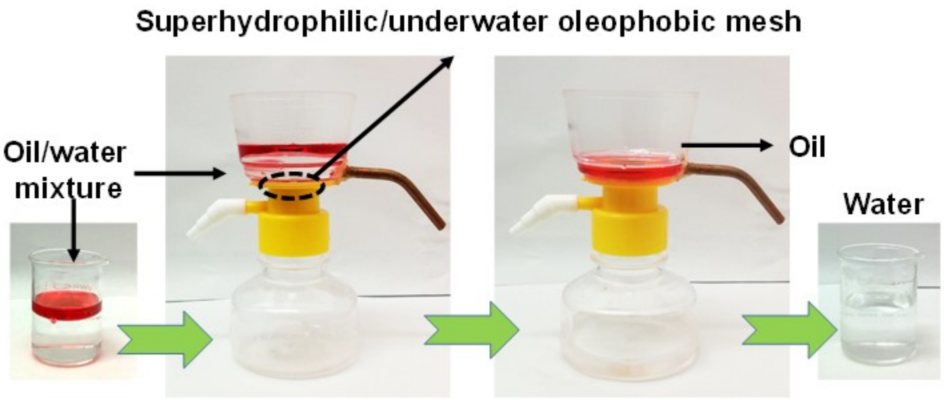

Figure S9. Experimental set-up for oil/water separation experiments.

Table S2 Experimental and theoretical results of Oil intrusion pressure of nanowires-haired membranes **[1]**.The estimated pore size was taken from [2].

**Mesh number**	**H_{max} (cm)**	**Intrusion pressure**	**Estimated pore size (µm)**	**H_{max} (cm)**	**Intrusion pressure**
	**Experimental results**			**Theoretical results**	
200	31	2.0	74	33	2.1
250	40	2.6	63	39	2.5
300	51	3.4		-	-
350	62	4.1		-	-
400	70	4.6	37	67	4.3

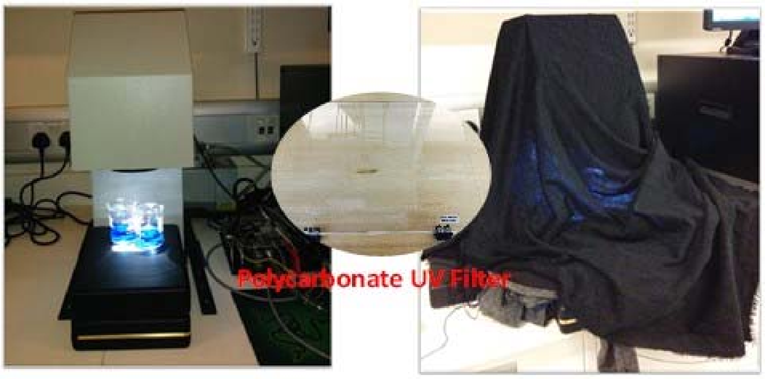

Figure S10. Experimental setup for illumination of samples using solar simulator.

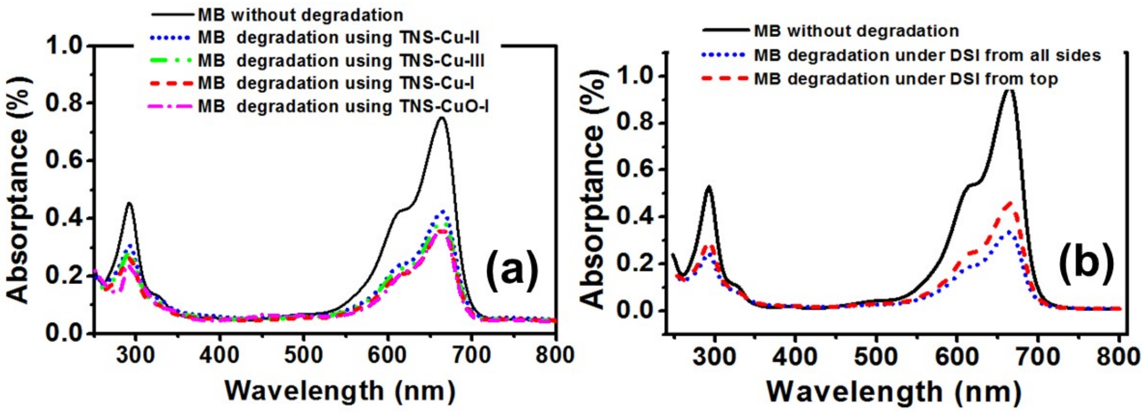

Figure S11. (a) The spectral absorptance of MB(aq) degradation using TNS-Cu-I, TNS-CuO-I TNS-Cu-II, and TNS-Cu-III meshes under 1 SUN irradiation using solar simulator for 2 h. (b) and The spectral absorptance of MB(aq) degradation using TNS-Cu-III under direct sun light irradiation for 2 h, respectively.

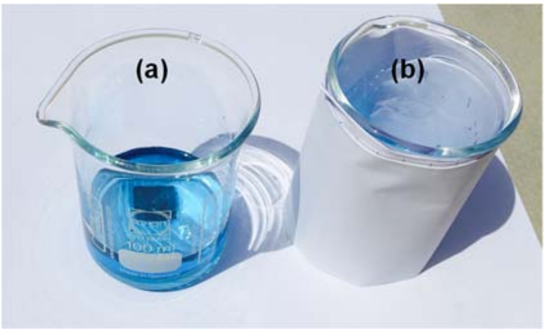

Figure S12. MB degradation under DSI (400 Wm^{-2}) on rooftop. (a) Uncovered and (b) covered from side walls.

References

1. Zhang, F., et al., *Nanowire‐Haired Inorganic Membranes with Superhydrophilicity and Underwater Ultralow Adhesive Superoleophobicity for High‐Efficiency Oil/Water Separation.* Advanced Materials, 2013. **25**(30): p. 4192-4198.
2. http://aprilma.sell.curiousexpeditions.org/pz5402f0a-china-welded-copper-mesh-copper-washed-standard-industrial-mesh-welded-copper-mesh-panels.html
3. Yuan, S., et al., *Biocorrosion behavior of titanium oxide/butoxide-coated stainless steel.* Journal of The Electrochemical Society, 2008. **155**(5): p. C196-C210.
